# Upper limb principal arteries variations: A cadaveric study with terminological implication

**DOI:** 10.17305/bjbms.2020.4643

**Published:** 2020-11

**Authors:** Marek Konarik, Vladimir Musil, Vaclav Baca, David Kachlik

**Affiliations:** 1 Department of Anatomy, Second Faculty of Medicine, Charles University, Prague, Czech Republic; 2 Department of Histology and Embryology, Third Faculty of Medicine, Charles University, Prague, Czech Republic; 3 Centre of Scientific Information, Third Faculty of Medicine, Charles University, Prague, Czech Republic; 4 Department of Health Care Studies, College of Polytechnics Jihlava, Jihlava, Czech Republic

**Keywords:** Anatomical variant, anatomical variation, axillary artery, brachial artery, radial artery, ulnar artery, anatomical terminology, anatomical nomenclature

## Abstract

Although the variability of the upper limb arteries is a clinically important problem, the prevalence is varying across the existing studies and classification is rather complicated, not well established and sometimes even unclear for simple and direct understanding and usage. Multiple case reports appearing in the last years apply incorrect, inappropriate, and sometimes misleading terminology. We performed an anatomical cadaveric study of the variability of the arteries of the upper limb, namely, the axilla, arm, and forearm, in 423 upper limbs embalmed with classical formaldehyde method (Central European population). We proposed to apply the Equality system based on the common trunks for denomination of the axillary artery branches principal variations: *Truncus subscapulocircumflexus* (22.9%), *truncus profundocircumflexus* (13.75%), and *truncus bicircumflexus* (13.95%). Further, we proposed the terminology system developed by Rodríguez-Niedenführ et al. for the free upper limb principal arterial trunk variations based on the origin, location (in the arm only, or in the arm and forearm), and course (related to the forearm flexor muscles) of the involved artery: *Arteria brachialis superficialis* (9.5%), *arteria brachioradialis superficialis* (6.4%), *arteria brachioulnaris superficialis* (1.9%), *arteria brachiomediana superficialis* (0.5%), and *arteria comitans nervi mediani manus* (3.3%). Extensive development of the catheterization methods via the *arteria radialis et ulnaris* as well as surgical procedures using flaps based on perforating branches of these arteries (including *arteria brachioradialis superficialis et brachioulnaris superficialis*) necessitate thorough data on prevalence of the variant vessels for safe performance of these procedures to prevent any unexpected situations or to react adequately in such.

## INTRODUCTION

**FIGURE 1:**
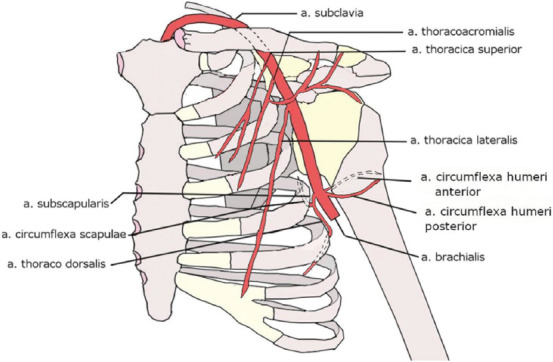
Textbook (usual) pattern of the *arteria axillaris* branching.

The variability of the upper limb arteries is a topic that can seem discussed, exhausted, and solved at first look (Figure 1). There exist many classical works and studies ranging from the middle of the 19^th^ century to the beginning of the new millennium [1-19], and many case-reports (not referred here) appearing especially in the past 15 years in Indian journals which both describe in different extent and detail the variational anatomy of the principal longitudinal arteries of the upper limb as well as the trunk variability of the axillary artery.

Most of the works published are comprehensive but a bit opaque and classify the variations in a rather complicated and/or obsolete way, sometimes even unclearly for simple and direct understanding of the reader. Similar problem comes with the case reports, often applying incorrect, inappropriate, and sometimes misleading terminology.

Our goal was to perform an extensive and thorough study in the Central European population which still lacks such results and to compare our data with works of the other authors. The outcome of the study should bring a simplification of the results as well as clear and simple variational terminology of the upper limb arteries for clinicians. The need of the knowledge of the clinical anatomy rises due to massive development of microsurgical and radiological methods.

## MATERIALS AND METHODS

During the past 12 years (from 2008 to 2019), we performed an anatomical cadaveric study of the variability of the upper limb arteries, namely, the axilla, arm, and forearm. The dissections were performed using the material from different anatomical institutions in the Czech Republic – Charles University in Prague and its five medical faculties (First Faculty of Medicine, Second Faculty of Medicine, Third Faculty of Medicine, Medical Faculty in Hradec Kralove, and Medical Faculty in Pilsen), Palacky University in Olomouc – and in Slovakia – Pavel Jozef Safarik University in Kosice and Comenius University in Bratislava. Altogether, we have dissected 423 upper limbs embalmed with classical formaldehyde method (52% right, and 48% left). Due to different conditions, there was not always possible to distinguish the sex of the specimen and that is why the gender data were excluded from the results.

## RESULTS

**FIGURE 2:**
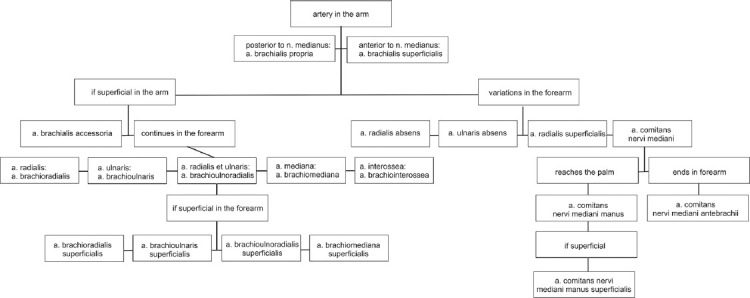
Terminological system of the upper limb principal arterial trunks proposed by Rodríguez-Niedenführ et al. in 2003 [17].

Within the axilla and arm, we found eight different principal variations, within the arm and forearm nine principal variations, and another five were restricted to the forearm only. The arteries of the wrist and hand were not included in our study. Tables 1-3 summarize the results of our study and present the other goal of our study – a precise terminology of the arterial variants. The system of applied terms is based on a nomenclature established by Rodríguez-Niedenführ et al. in 2001 [16], which covers all the longitudinal principal variants which can appear, including also the theoretical (yet not reported) possible patterns (Figure 2). Following paragraphs are devoted to each principal variation in detail.

**TABLE 1:**
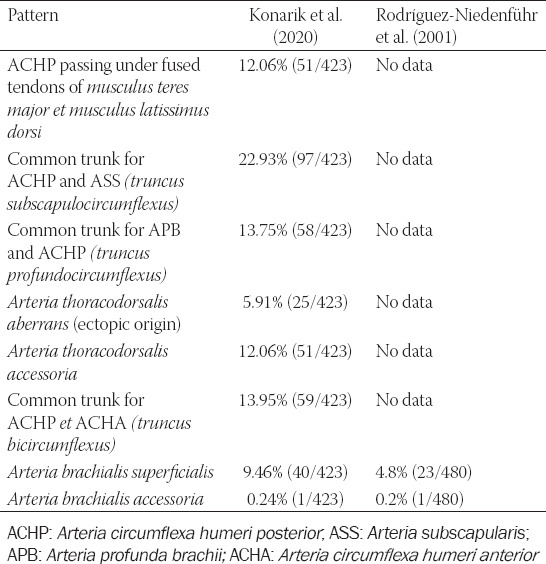
Variable principal arteries in the axilla and arm

**TABLE 2:**
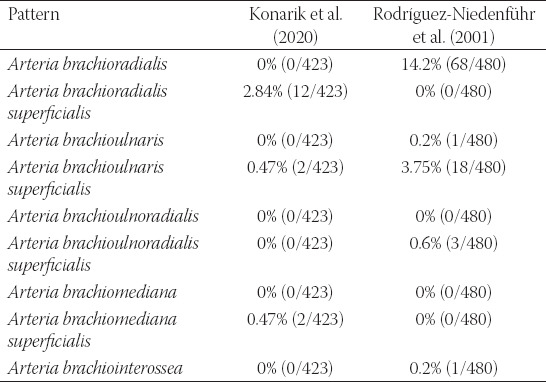
Variable principal arteries in the arm and forearm

**TABLE 3:**
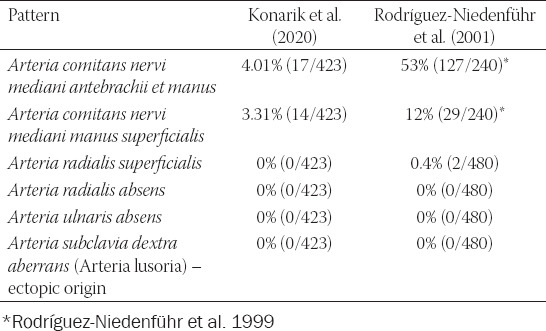
Variable principal arteries in the forearm only plus *arteria subclavia* main variant

### *Arteria circumflexa humeri posterior* (ACHP) passing under the fused tendons of the *musculus teres major* and *musculus latissimus dorsi*

We found this unusually running ACHP in 12.06% (51/423) of cases. The standard course of the ACHP is defined as an artery branching from the *segmentum infrapectorale arteriae axillaris*, running dorsal to the humerus together with the axillary nerve, entering the *foramen humerotricipitale* (quadrilateral space) [18] to supply the *articulatio humeri* and *musculus deltoideus*.

The variant is defined as coursing underneath the fused tendons of the *musculus teres major* and *musculus latissimus dorsi* which are together inserted to the *crista tuberculi minoris*. Then, the variant ACHP turns behind the tendons and ascends dorsal to them to its usual area of supply. Thus, it ­features a longer course than the proper ACHP and is often combined with another variation stated below (*truncus profundocircumflexus*). This variation was reported by many authors [1,3,5-7,9,11,12,14-16,19-28] but only minority of them brought the prevalence (Table 4).

**TABLE 4:**
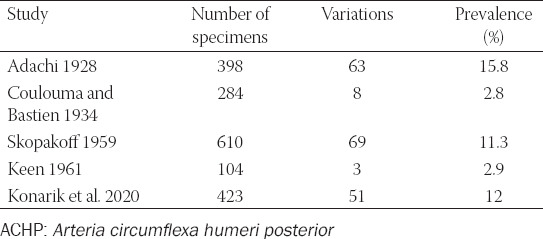
Overview of studies concerning the prevalence of the ACHP passing under the fused tendons of the *musculus teres major* and *musculus latissimus dorsi*

### Common trunk for *arteria circumflexa humeri posterior* and *arteria subscapularis* (*truncus subscapulocircumflexus*)

We observed this variable trunk in 22.93% (97/423) of cases which ranks it the most common variation in our study. The ACHP is usually a branch from the *segmentum infrapectorale arteriae axillaris* but the *arteria subscapularis* (ASS) originates more proximally, usually from the *segmentum retropectorale arteriae axillaris*. In this pattern, the ACHP and ASS originate from a short common trunk (*truncus subscapulocircumflexus*), located usually at the transition of both segments, i.e., at the level of the inferior border of the *musculus pectoralis minor*.

This variation was reported by following authors: [1,3,5,6-9,11,12,14-16,19-28] but only some reported on its prevalence (Table 5). Pestemalci et al. (1999) found the variation in 32% cases [29], on the contrary De Garis and Swartley (1928) presented opposite result (only 1.4%) [5]. Adachi (1928) stated that ACHP stems directly from the *arteria axillaris* (AA) in 33% only which means that its origin is aberrant (ectopic) in 67%, i.e., in 39.7% as *truncus subscapulocircumflexus* and in 30.3% cases as *truncus profundocircumflexus* from the *arteria profunda brachii* (APB) – see below [6].

**TABLE 5:**
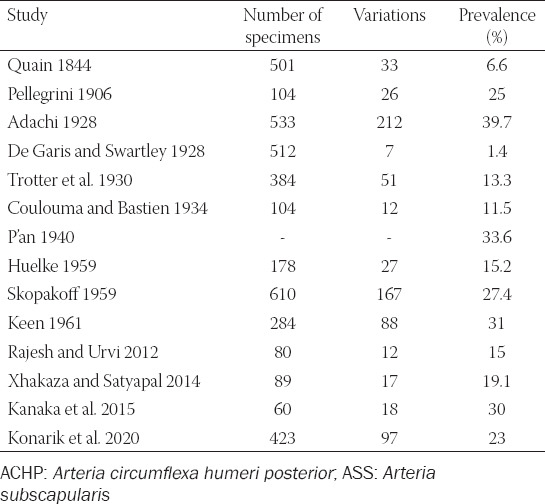
Overview of studies concerning the prevalence of the common trunk for the ACHP and ASS (*truncus subscapulocircumflexus*)

### Common trunk for *arteria circumflexa humeri posterior* and *arteria profunda brachii* (*truncus profundocircumflexus*) (Figure 3)

**FIGURE 3:**
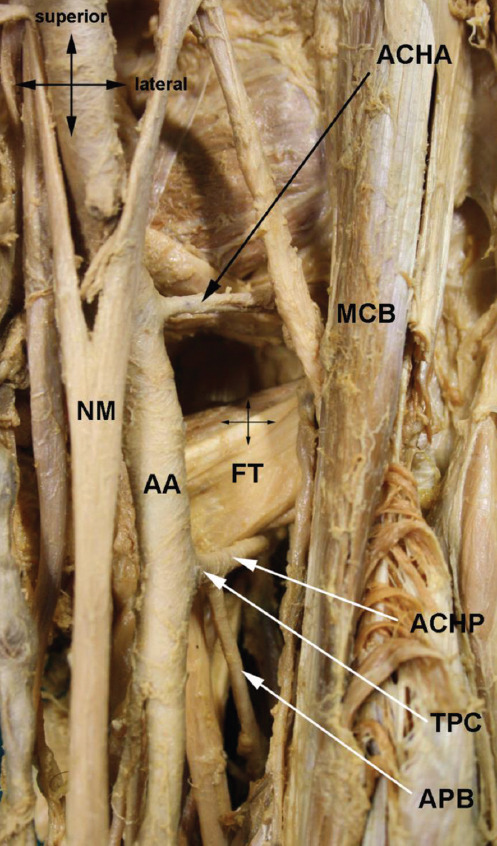
*Truncus profundocircumflexus*. AA – *arteria ­axillaris*; ACHA – *arteria circumflexa humeri anterior*; ACHP – *arteria circumflexa humeri posterior*; APB – *arteria profunda brachii*; FT – *fused tendons of musculus teres major and musculus latissimus dorsi*; MCB – *musculus coracobrachialis*; NM – *nervus medianus*; TPC – *truncus profundocircumflexus*.

We registered this variable trunk in 13.75% (58/423) of cases. The APB usually branches from the proximal segment of the *arteria brachialis* a few centimeters below the inferior margin of the fused tendons of the *musculus teres major* and *musculus latissimus dorsi*, the ACHP a bit more proximal from the *segmentum infrapectorale arteriae axillaris*. The common trunk (*truncus profundocircumflexus*) is short and thick. Its arrangement can be described as three different types, depending on the caliber of the trunks and its terminal branches (ACHP and APB), as described in some studies (Table 6). The most common type features the larger ACHP and it can imply that APB is a smaller branch from an aberrant ACHP. This variation was reported by several authors [1,5,6,11,12,21-23,30,31].

**TABLE 6:**
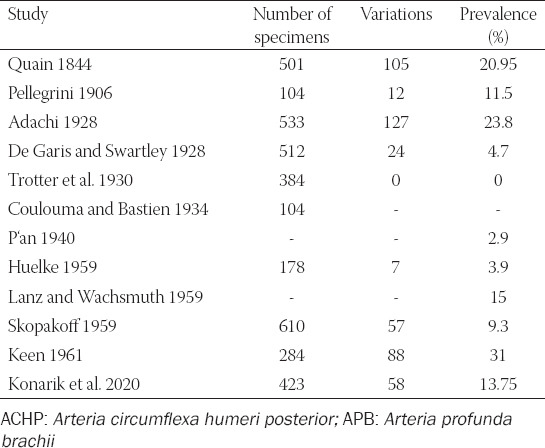
Overview of studies concerning the prevalence of the common trunk for the for the ACHP and APB (*truncus profundocircumflexus*)

#### Arteria thoracodorsalis aberrans

We found this unusually originating artery in 5.91% (25/423) of cases. The *arteria thoracodorsalis* usually stems from the short and thick *arteria subscapularis* as a terminal branch from its bifurcation (the other branch is the *arteria circumflexa scapulae*). Its course is firmly constant unlike its origin, which can often vary. We have found two sites of aberrant (ectopic) origin: *Arteria thoracica lateralis* and *arteria thoracoacromialis*. The same case has also been presented by Maral et al. (1993) [32]. Huelke (1959) found cases with the *arteria thoracica lateralis* stemming from the *arteria thoracodorsalis* or ASS (together in 28.7%) but the case of the aberrant *arteria thoracodorsalis* only in 0.7% [23]. Trotter et al. (1930) published similar results (25%) [21] to Huelke, on the contrary, Adachi (1928) reported the variation in 8.3%, similar to our results [6].

#### Arteria thoracodorsalis accessoria

We observed this supernumerary (accessory) vessel in 12.06% (51/423) of cases which means a presence of two *arteriae thoracodorsales* supplying the same target muscle (*musculus latissimus dorsi*). The *arteria thoracodorsalis propria* stemmed from the ASS and supplied the *musculus latissimus dorsi*, running along the homonymous nerve. The accessory artery originated from the ASS as well or directly from the *arteria axillaris* or its other branches. According to the previous studies, it is a very rare finding as we have found only case reports [33-35].

### Common trunk for *arteria circumflexa humeri posterior* *et* *anterior* (*truncus bicircumflexus*)

**FIGURE 4:**
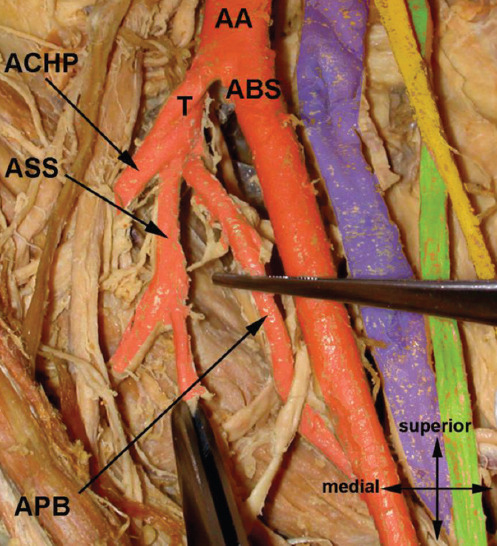
*Arteria brachialis superficialis and truncus profundosubscapulocircumflexus*. Legend: AA – *arteria axillaris*; ABS – *arteria brachialis superficialis*; ACHP – *arteria circumflexa humeri posterior*; APB – *arteria profunda brachii*; ASS – *arteria subscapularis*; T – *truncus profundosubscapulocircumflexus*.

We registered this variable trunk in 13.95% (59/423) of cases. The *arteria circumflexa humeri anterior* usually originates at the same level as the ACHP but is thinner and heads ventrolaterally to contribute to the supply of the *caput humeri* and *articulatio humeri* as well as the *musculus deltoideus*. The common trunk (*truncus bicircumflexus*) was first mentioned by Meckel (1839) [36] and is not often reported in literature: Quain (1844) – 6%; Hitzrot (1901) – 16%; Pellegrini (1906) – 22%; and Poynter (1920) – 20% [1,20,30,37]. Piersol (1919) emphasized in his work that the *truncus bicircumflexus* can be combined with the ASS as *truncus subscapulobicircumflexus* or also with the APB as *truncus profundosubscapulobicircumflexus* [38] (Figure 4); Saeed et al. (2002) reported such trunk in 3.8% of cases [39] and Rajesh and Urvi (2012) in 15% of cases [40]. These authors even reported a common trunk for ACHP, PCHP, ASS, and APB (*truncus profundosubscapulobicircumflexus*) in 10% of cases.

#### Arteria brachialis superficialis

We found this variation in 9.46% (40/423) of cases. This is a special kind of variation including the course of a longitudinal arterial trunk. The *arteria brachialis* courses usually deep (dorsal) to the *nervus medianus*. In case it gets superficially at the caudal end of the *fossa axillaris*, it descends ventral to the *nervus medianus* as far as the *fossa cubitalis* to divide into the *arteria radialis* and *arteria ulnaris* as usual [13,24].

#### Arteria brachialis accessoria

We observed this supernumerary vessel in 0.24% (1/423) of cases. If it is present, there are two main longitudinal stems descending along the arm – *arteria brachialis* (in this case often described as *arteria brachialis propria*) in its usual position and *arteria brachialis accessoria*, usually coursing ventral to the *nervus medianus*. The latter joins the former within the *fossa cubitalis* (rarely more proximal) just before the final bifurcation into the *arteria radialis et ulnaris*. It is a very rare variant, described as case reports only [9,41-44].

#### Arteria brachioradialis superficialis

We registered this variation in 6.38% (27/423) which ranks it the most common variation of the arm and forearm longitudinal arterial trunks in our study as well as in other cadaveric studies [1,3,6,9,11,12,15,16,45-50] – as well as radiographic or ultrasound studies – [25,51-58] – although Zhan et al. (2010) reported only two cases out of 1200 limbs examined in Singapore Chinese population [48]. Based on Lippert and Pabst (1985) review [14], it is present in 8% (3% with cubital anastomosis) but the prevalence data differ a lot (Table 7).

**TABLE 7:**
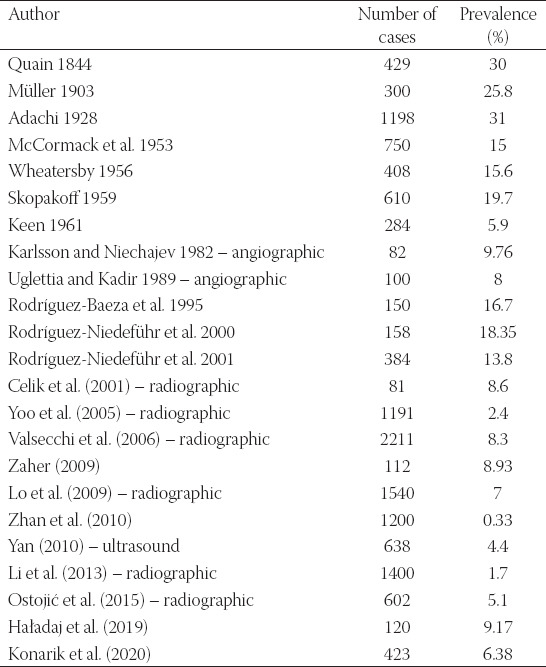
Overview of studies concerning the prevalence of the *arteria brachioradialis superficialis*

The *arteria radialis* originates from the terminal bifurcation of the *arteria brachialis* within the *fossa cubitalis*. If the origin is shifted proximally to the *fossa cubitalis*, it is colloquially called “radial artery with high origin” but the more appropriate term is *arteria brachioradialis*. Based on the relationship to the forearm flexors, we can further distinguish superficially coursing *arteria brachioradialis* *superficialis* which takes majority of this variant, and deeply running *arteria brachioradialis* which is extremely rare and was not observed in our study. However, the majority of the authors does not concern about the level of the course and does not unfortunately specify it with the adjective “*superficialis*.”

#### Arteria brachioulnaris superficialis (Figure 5)

**FIGURE 5:**
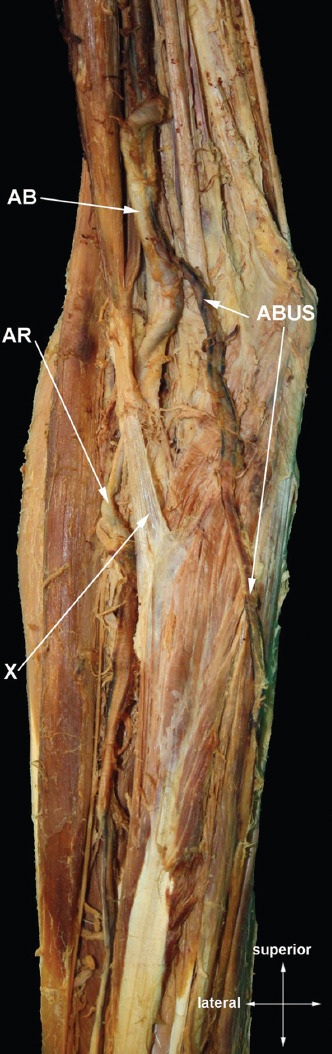
*Arteria brachioulnaris superficialis*. AB – *arteria brachialis*; ABUS – *arteria brachioulnaris superficialis*; AR – *arteria radialis*; X – *aponeurosis bicipitalis*.

We found this variation in 1.89% (8/423) of cases. It is similar situation to the variant described right above, i.e., the *arteria ulnaris* originating proximally to the *fossa cubitalis*. In case it courses superficial to the forearm flexors, it is called *arteria brachioulnaris superficialis*, while in the opposite case it is called *arteria brachioulnaris* which is extremely rare and was not observed in our study. It is quite known artery, reported in many cadaveric studies [5-7,9,11-13,16,19,22,41-43,45,59-69] as well as surgical and angiographic [25,51,63,68-70] (Table 8).

**TABLE 8:**
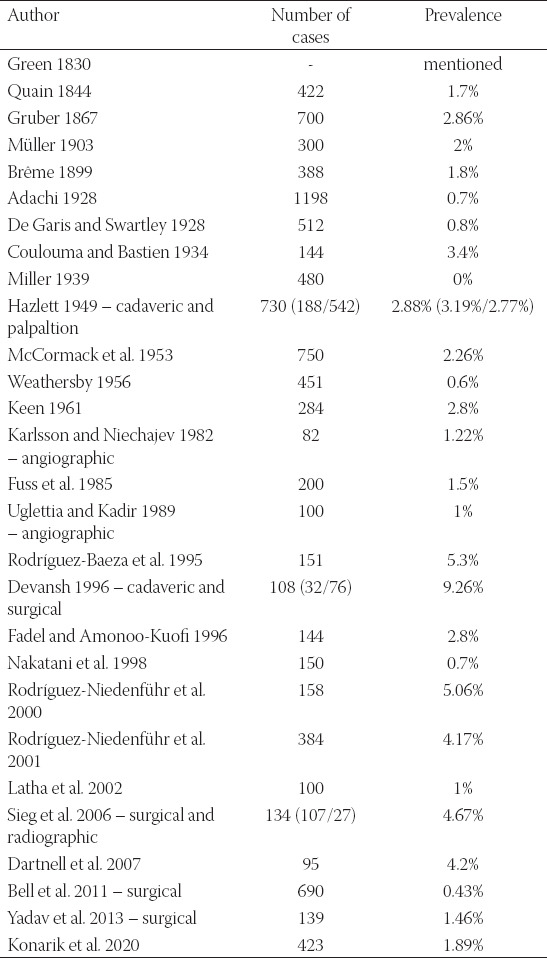
Overview of studies concerning the prevalence of the *arteria brachioulnaris superficialis*

#### Arteria brachiomediana superficialis

We observed this variation in 0.47% (2/423) of cases. It is a longitudinal arterial trunk accompanying the *nervus medianus* not only in the forearm (see below) but also in the arm. In all described cases it continued as far as the hand through the *canalis carpi* [1,6,9,14,71,72-77] (Table 9). In case it courses superficial to the forearm flexors, it is called *arteria brachiomediana superficialis*; in the opposite case it is called *arteria brachiomediana* which is extremely rare and was not observed in our study.

**TABLE 9:**
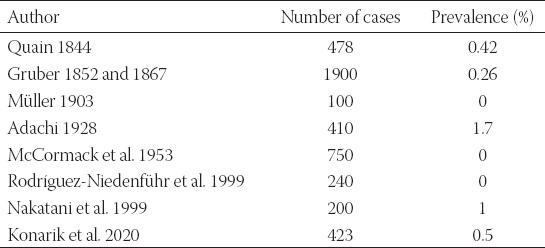
Overview of studies concerning the prevalence of the *arteria brachiomediana superficialis*

#### Arteria brachioulnoradialis superficialis

We did not observe this variant in our study. It is the *arteria brachialis superficialis* bifurcating into its usual two terminal branches but unusually proximal to the *fossa cubitalis* and its branches always run superficial to the forearm flexors as reported in several studies [1,12,13,15,16,78-86]. The purely theoretical variant with terminal branches coursing deep to the forearm flexors would be called *arteria brachioulnoradialis*.

#### Arteria brachiointerossea

Similar to the previous item, we did not observe this variant in our study. It is the *arteria interossea* branching unusually proximal to the *fossa cubitalis* and thus not from the *arteria ulnaris* but from the *arteria brachialis*. It always courses deep to the forearm flexors but its occurrence is quite rare [16,87-90]. It is necessary to distinguish this variant from the previous – *arteria brachioulnoradialis superficialis* – in which case the deeply located trunk (*arteria brachialis*) continues in its usual course into the *arteria interossea communis* [85].

#### Arteria comitans nervi mediani antebrachii et manus superficialis

We registered this variation in 9.9% (42/423) and 3.3% (14/423) of cases, respectively. It is a longitudinal arterial trunk accompanying the *nervus medianus* in the forearm and can continue into the hand through the *canalis carpi*. It originates from the *arteria ulnaris, arteria interossea communis*, or *arteria interossea anterior* and is of various calibers. The variability of its caliber and length is the cause of very differing data presented in published studies [1,4-7,14-17,75,91-98]. As we concentrated on the larger (principal trunks) of the upper limb, we have paid attention only to those larger than 1 mm in the distal forearm and further comparisons go beyond the scope of this study.

Generally, the vessel accompanying the *nervus medianus* can exist in five different types:

- It originates in the arm and courses superficial to the forearm flexor muscles (*arteria brachiomediana superficialis*) – quite rare
- It originates in the arm and courses deep to the forearm flexor muscles (*arteria brachiomediana*) – theoretical
- It originates in the forearm and terminates distally within the forearm (*arteria comitans nervi mediani antebrachii*) – common
- It originates in the forearm, courses deep to the *retinaculum musculorum flexorum* through the *canalis carpi* and terminates in the hand (*arteria comitans nervi mediani manus*) – rare
- It originates in the forearm, courses superficial to the *retinaculum musculorum flexorum* outside the *canalis carpi* and terminates in the hand (*arteria comitans nervi mediani manus superficialis*) – extremely rare.

Very often the term *arteria mediana*/*arteria mediana persistens* is used but we consider this term appropriate for the transient embryological vessel and if it persists to the adult age it should be distinguished and considered as a distinct unit – *arteria comitans nervi mediani*. The discussion concerning this variant and its terminology goes beyond the scope of the article.

## DISCUSSION

The textbook/usual branching of the *arteria axillaris* appears only in the minority of the population which is a long known fact. Adachi (1928) classified 34 different variations of the axillary artery [6], Lippert and Pabst (1985) summarized the variability of the axillary artery in 90% of cases [14]. The most common variations concern the ASS, ACHP, and APB [1,6,9,11,14,21-23,27,28,71,72,74,85,101-109,70,72,83,98-104].

The variability of the upper limb principal arteries was present in 77% of cases (328/423). Inspired by the work of Feigl and his team (2012) [110], we have checked all the individual investigations (21), performed separately at different departments (8), and in different year (12) to see if the small number of investigated specimens could affect the prevalence. Using the example of the *truncus profundocircumflexus* (Table 10), we support their conclusion that a large number of specimens is the only guarantee to bring reliable prevalence data.

**TABLE 10:**
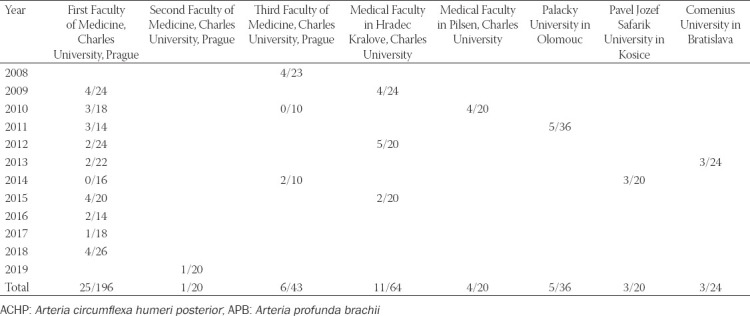
Findings of the common trunk for ACHP and APB (*truncus profundocircumflexus*) in individual dissection procedures (total number: 58/423; prevalence: 13.75%)

The main and striking problem which we faced during our study and while comparing our data with the literature sources, was missing official nomenclature and inconsistent usage of more synonyms without their prior and appropriate definition. There exists no official variant anatomy, and Terminologia Anatomica, the last edition of the official anatomical nomenclature, contains only 31 items in the chapter *Systema cardiovasculare* and only one item concerning the arteries of the upper limb (A12.2.09.019 = *a. brachialis superficialis*) [111]. Thus, the authors apply various terms based on their geographical location, language knowledge, and tradition, especially in case reports and older studies. The only consistent and comprehensive terminology was proposed in 2001 by Rodríguez-Niedenführ and his team [17] (Table 11) – see Figure 2.

**TABLE 11:**
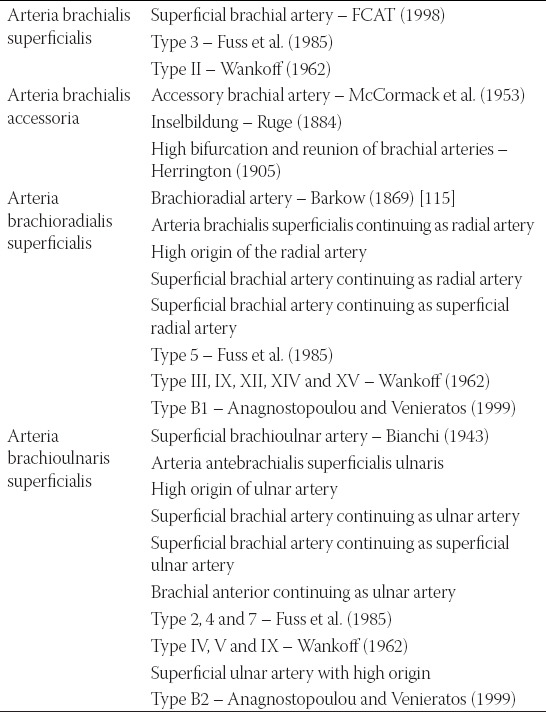
The synonyms existing to the terminology proposed by Rodríguez-Niedenführ et al. in 2001 [89]

Another problem which appeared during the sources review was different and subjective sorting of the variations into groups, especially concerning the *arteria axillaris*, and some of them used a too detailed and complicated classifications not easily translatable into clinical practice.

There exists no general rule for definition of the variant vessels, especially concerning their unusual (aberrant/ectopic) origin. If the artery arises from an unusual site of the same parent artery or from another artery (either daughter artery or parallel/collateral artery), it should be called “aberrant.” Moreover, there appear two approaches for its specific description:

1. Subordination system – aberrant artery is a branch from another constant artery (e.g., ACHP is usually a branch from the *segmentum infrapectorale arteriae axillaris* but variably it can originate either proximally from other segments or from daughter branches of the *arteria axillaris*, most commonly from the ASS, or distally from the *arteria brachialis*, or its daughter branch APB)
2. Equality system – both arteries are equal and arise from a common trunk, based proximally to their origins (e.g., ACHP can originate from common trunks with other daughter branches of either the *arteria axillaris* or *arteria brachialis*, most commonly from the *truncus subscapulocircumflexus* (with ASS), or *truncus profundocircumflexus* (with APP), respectively).

Already these two systems give a possibility to describe one variation in two different ways and thus to produce new terms (synonyms) for existing ones. This should be a strong motivation enough for constitution of a variant anatomical nomenclature in near future by a large team of specialists. We found six common different types of the principal arterial trunks within the *fossa axillaris* and even despite of the extensive number of dissected limbs, we did not encounter all the variations described by the previous authors.

If we go back to the terminology of the free upper limb principal arterial trunk variations, Rodríguez-Niedenführ and his team came with a very sophisticated system of the nomenclature of the variations distal to the axillary artery end [17]. Their method uses designation of the origin, location (in the arm only, or in the arm and forearm), and course (related to the forearm flexor muscles) of the involved artery. As an example, we can talk about a variable artery that stems from the *arteria brachialis* proximal to the *fossa cubitalis* and courses along the medial side of the forearm superficially to the forearm flexor muscles. Functionally, this artery replaces the *arteria ulnaris*, but based on its beginning and course it cannot be described as the *arteria ulnaris*. When applying the above mentioned method, it should be called the *arteria brachioulnaris superficialis*.

Unfortunately, this method cannot be applied to the nomenclature of the *arteria axillaris* branches variations that is why we decided to use the Equality system described above which is based on common trunks. As an example, we can talk about *arteria circumflexa* *humeri anterior et posterior* which differ by caliber (the former is thinner) but if they share a common origin, we propose the term *truncus bicircumflexus* (Table 1).

## CONCLUSION

Last years brought an extensive development of the catheterization methods via the *arteria radialis* and *arteria ulnaris* as well as surgical procedures using flaps based on perforating branches of these arteries (including *arteria brachioradialis superficialis et brachioulnaris superficialis*) [112-114]. Any detailed anatomical studies bringing thorough data on the prevalence of the variant vessels are of utmost importance for clinicians performing these procedures to prevent any unexpected situations or to react adequately in such. Moreover, unanimous and clear nomenclature is an easy communication tool for everybody involved in diagnostic, therapeutic, and education processes.
